# Family routines within the ecological niche: an analysis of the psychological well-being of U.S. caregivers of children with disabilities

**DOI:** 10.3389/fpsyg.2014.00495

**Published:** 2014-05-30

**Authors:** Elizabeth Larson, Thomas Miller-Bishoff

**Affiliations:** Occupational Therapy Program, Department of Kinesiology, University of Wisconsin–Madison, MadisonWI, USA

**Keywords:** developmental disability, family, routines, psychological well-being, parenting, eco-cultural niche

## Abstract

Using mixed methods, this study examined the relationship of caregivers of children with disabilities’ psychological well-being (PWB) and their orchestration of daily routines within their ecological niche. Thirty-nine U.S. caregivers completed in-depth interviews, *PWB Scales*, and *Family Time and Routines Index (FTRI*). We used a multi-step analysis. Interview data was coded and vignettes created without knowledge of *PWB* and *FTRI* ratings. Next, the relationship of quantitative measures was analyzed. Four groups were created using *FTRI-extent* and *PWB* means: (1) low routine-low *PWB*, (2) low routine-high *PWB*, (3) high routine-low *PWB*, and (4) high routine-high *PWB.* We examined qualitative differences in key features between groups. *Findings*: Total *PWB* and *FTRI* scores were not significantly correlated, *PWB Purpose in Life* and *FTRI-extent* scores were moderately positively correlated, and *PWB Environmental Mastery* and* FTRI-extent* correlation approached significance. Qualitative findings describe caregivers’ structuring of routines, intensity of oversight, support in routines, management of dinner, paid work, and needs for respite. The four groups differed in paid work, household support, degree the child could self-occupy, *Environmental Mastery,* and opportunities to recuperate. Caregivers with higher levels of well-being and more regular routines did paid work, had supportive spouses, had children who more often could follow routines, had higher *Environmental Mastery*, could orchestrate a family meal, and had breaks from care in either work or leisure. All Native American caregivers and Mexican American caregivers with spouses were in the high routine-high *PWB* group. Insight into this complex negotiation between family members within daily routines may provide practitioners a better understanding of how to work within family circles to foster therapeutic alliances, identify focused intervention targets, and promote positive family wide outcomes.

## INTRODUCTION

The “production” of family routines often relies on one key individual – usually the mother – to create the architecture of the schedule and to orchestrate and tune the execution of these daily practices to meet the family members’ current and emerging desires and needs (Larson, 1996). Family routines provide structure, a sense of security, and opportunities for emotional connection (Rodger and Umaibalan, 2011; Crespo et al., 2013). For families of children with disabilities, meaningful routines have been shown to be more difficult to establish and more utilitarian in function (Lucyshyn et al., 2004; Rodger and Umaibalan, 2011). These alterations were due to disruptions related to the child’s needs, a lack of needed resources, and time pressures related in part to juggling demands for the child’s care and therapies (Larson, 2006; Fiese, 2007; Myers et al., 2009; Sawyer et al., 2010; Rodger and Umaibalan, 2011; Schaaf et al., 2011). Thus the incorporation of interventions that change routines, as recommended by health professionals or educators, may require greater maternal efforts to establish, and may be resisted if not aligned with the family’s overall desires and goals. Adherence to principles of family-centered practice compels us to recognize the investments caregivers must make to institute therapeutic activities within daily routines and the impact these changes may have on the family’s quality of life.

Routines are organized, sequenced activity patterns that occur at specific time and in specific space. This tight and intricate sequencing allows the family to organize the individual and shared activities necessary to sustain health, well-being and connectedness among family members. Routines provide stability and predictability to daily life yet are dynamic, shifting as the child’s abilities develop and altering across the developmental stages of the family. Typically, the cycles move from a centripetal state with family life organized around the young child to a centrifugal state at school age, when there is a move away from intense caretaking demands (McCubbin et al., 1996). This shift, however, may not occur in the same way for families of children with disabilities due to the child’s slower progress or prolonged caregiving demands (Birenbaum, 1971; Combrinick-Graham and Higley, 1984). Mothers may not only have to engage in greater intensity of caregiving over time but they may also not experience the same emotional rewards as parents of typical children (Roberts, 1984).

Like habits, routines may not be innately nor essentially good. Cultural values, expectations, and perspectives are passed on across generations predominantly at the level of the family unit (Bourdieu, 1990), and family routines and rituals are key to this transmission (Fiese et al., 2002). For the family, the daily routines’ sustainability and “goodness” may depend largely upon subjective judgments of the degree to which the current routines: (1) meet the family goals, (2) are sensitive to the motivation and emotions of participants, (3) address core tasks such as provision of food and clean clothing, and (4) foster appropriate “normative” participation directing who and how an activity should be done (Weisner, 1997). According to the ecocultural theory of development, culture is visible and lived in the practice of everyday routines (Weisner, 1997). Ecocultural theory is a useful frame for considering the relationship of caregiver well-being and routines since it considers the intersection of demands, cultural scripts, values, resources, and barriers and how these are negotiated on a daily basis within family routines (Gallimore et al., 1989). Caregiver roles and responsibilities, children’s autonomy, age- and gender-related expectations, and attitudes toward disability are all related to family’s cultural values (Spagnola and Fiese, 2007; Huang et al., 2011; Desai et al., 2012).

Family routines occur within and are supported by features of the family’s ecological niche, their surrounding socio-cultural contexts (Bronfenbrenner, 1986; Bernheimer and Weisner, 2007). The niche includes features such as family subsistence, accessibility of health and education services, home and neighborhood safety and convenience, domestic task workload, child care tasks, marital role relationships, father’s role in child care, social support and sources of parental information (Gallimore et al., 1989; Bernheimer and Weisner, 2007). In their study of white families of children with developmental disabilities, Gallimore et al. (1989) found parents created accommodations related to the “hassle level” of the child in niche features of subsistence, health and intervention services, child safety and domestic workload.

While the features of each family’s niche are unique, as are their adaptations, research suggest trends in ways these features support, as well as challenge, the caregiver’s management of family routines. In terms of subsistence, research shows the majority of caregivers remain home allowing them to assure the quality of their child’s care. Only one-quarter to one-third of caregivers of children with severe disabilities remained employed outside the home (Thyen et al., 1999; Curran et al., 2001). This appreciably reduced family income while the child’s care expenditures increased, although wide variations have been reported in employment and care costs (Thyen et al., 1999; Curran et al., 2001; Olsson and Hwang, 2006; Anderson et al., 2007). In regards to provision of services, caregivers may not always feel supported by the local systems of service provision. Mothers reported spending significant time negotiating within the health and education systems to assure their child’s needs were met (Murphy et al., 2006). One-quarter of 121 families of children with disabilities rated their primary care physician as fair or poor at understanding the impact of the child’s disability on family life (Liptak et al., 2006). The most dramatic unmet need clearly affects caregivers; three-quarters of families of children with disabilities had unmet needs for respite care (Rupp et al., 2005-2006). These are just a few examples of how the ecocultural niche features may affect family routines and place demands upon the caregiver who orchestrates them.

Caregivers’ health and well-being may be negatively impacted by limits to and stress within their daily routines. Mothers of children with disabilities experienced more stress related to daily routines than mothers of typically developing children (O’Brien, 2004). Accommodations for the child may include creating more predictable and narrow family routines that limit spontaneity, family outings, participation in parties and family rituals despite the value of these routines to the whole family (Lucyshyn et al., 2004; Larson, 2006). This refocusing and reshaping of routines can be overwhelming for caregivers (Hoogsteen and Woodgate, 2013). It may be certain qualities of routines rather than the amount of time spent caregiving that impacts caregiver’s well-being. In their study of 216 mothers of children with autism in Australia, Sawyer et al. (2010) noted a significant relationship between mental health problems and time *pressure* and no correlation between mental health problems and *total time* spent on caregiving tasks. Yet having too little time to manage routines may be problematic too.

While understanding daily life within families of children with disabilities may be integral to intervention and health promotion, few studies have considered the family ecology nor attended to differences in family scripts that may be driven by values of different ethnicities in the United States. Examinations of differences among the majority and minority populations in the U.S., for example European American, African American, and Hispanic, have focused on styles of parenting or interactions between typical children and their parents (e.g., Gibson-Davis and Gassman-Pines, 2010). Rarely have studies considered ethnic differences in families of children with disabilities. A recent study by Gannotti et al. (2013) did examine parent-child interactions in an ethnically diverse group of families of children with disabilities. Using an analysis of a large U.S. national sample, 24% of the variation in parents’ engagement in daily activities could be explained by the child’s socio-emotional skills and disability, family socioeconomic status, religious beliefs and ethnicity (Gannotti et al., 2013). The child’s social skills and behaviors contributed far more than ethnic differences in predicting daily parenting practices. Gallimore et al. (1989) posit that cultural places (e.g., United States or China) exert strong influences on construction of activity settings, shaping and influence the choices of activities of all families residing there despite the presence or absence of a family member with a disability. Yet they also argue that interesting and significant differences may be revealed by studying families whose children have unique developmental trajectories (Gallimore et al., 1989).

The research reviewed provides an understanding of the alterations of family routines when a child with disability is present, yet it is not clear what specific features of managing the daily routines are costly or beneficial to caregivers’ well-being, nor what contributions different ethnic backgrounds may add to well-being when parenting a child with a disability. Construction of daily routines is complex, multi-layered and driven by a range of motivations and beliefs as influenced by the larger socio-cultural context in which the family resides. To disentangle this complexity a mixed method approach is necessary: using available standardized measures to examine whether there is a relationship of routines to caregiver well-being, using a qualitative or generative approach to better understand key features of routines and how caregivers organize or manage daily routines, and finally integrating these qualitative–quantitative approaches to identify which caregivers fare better related to their orchestration and management of routines. This mixed method study explores the relationship between caregivers of children with disabilities’ negotiation and orchestration of daily routines within their U.S. ecological niches, and their self-rated psychological well-being (PWB).

## MATERIALS AND METHODS

### PARTICIPANTS

Forty-eight caregivers of children with disabilities volunteered for the study; thirty-nine caregivers completed all the measures used for this analysis. Purposive sampling was employed to ensure an ethnically- and geographically diverse sample. All participants lived in the United States, residing in the East coast, West coast or Midwest regions. Twenty-five participants self-identified as European American, five as Native American, seven as Mexican/Mexican American, one as Asian American and one as African American. We intentionally gathered a diverse group of caregivers that closely represent the diversity of a national sample CDC National Health Interview Survey (NHIS; 1999). The exceptions to this were an over-representation of Native American caregivers and insufficient representation of African American caregivers, whom we were less successful in recruiting.

Thirty-eight of the participants were female and one was male. Participants ranged in age from 26 to 50 years (*M* = 37.08 years, SD = 7.04 years). Thirty-four participants were married, three were divorced, and two were single. Yearly family income ranged from $9,600 to $200,000 (*M* = $53,876). Eighteen of the participants managed childcare and households full-time, 10 worked for pay part-time, eight worked for pay or went to school full-time, and three were on disability support or retired. Education ranged from sixth grade to a doctoral degree, with 29 participants having at least some college education. Their children ranged in age from 10 months to 17 years. Nine children had autism spectrum disorder, seven had cerebral palsy, seven had chronic medical conditions/medical fragility, five had developmental delays, seven had speech delays and/or dyspraxia, and four had other conditions including fetal alcohol syndrome, blindness, traumatic brain injury, and severe learning disability. Five families included multiple children with disabilities.

### MEASURES

#### Psychological well-being

Participants completed the 14-item version of Ryff’s *PWB Scales*. Using a six-point Likert scale anchored with “strongly disagree” to “strongly agree,” participants rated their PWB across six scales: *Autonomy, Environmental Mastery, Personal Growth, Positive Relations with Others, Purpose in Life,* and* Self Acceptance* (14 items per scale; Cronbach’s alphas between 0.83 and 0.91). The *PWB* scales were developed as a eudaimonic measure of well-being, examining dimensions that foster the individual’s capacity to thrive (Ryff, 1989). The *Autonomy* scale examines the degree the individual’s uses their own standards to guide their actions. The *Environmental Mastery* scale assesses the individual’s sense of competency to make use of opportunities they find valuable. On the *Personal Growth* scale the individual rates their self-improvement over time. The *Positive Relations* scale has an individual rate qualities of their relationships including the positivity, trust and intimacy. *Purpose in Life* assesses meaningfulness and goal directedness of life. Lastly, *Self-Acceptance* items assesses how positively a participant views herself or himself and her or his personal attributes. The total of all six scales was used as the measure of caregivers’ well-being in this analysis. Spanish-speaking participants received a Spanish translation of the scale (14 items per scale; α = 0.68 - 0.83). The *PWB* score is computed by summing scores of the six Ryff’s *PWB* subscales: *Personal Growth, Positive Relations with Others, Purpose in Life, Autonomy, Self Acceptance*, and *Environmental Mastery*. Participants with low literacy levels were offered the opportunity to complete this scale and the next one orally with a member of the research team. No participant accepted this offer.

#### Family routines

The *Family Time and Routines Index (FTRI*) was chosen as the measure to assess family routines because it assesses both the extent families practice certain routines and the meaning attached to these routines, and because it has comparison data (McCubbin et al., 1996). The *FTRI* is an expansion of Boyce’s *Family Routines Inventory (FRI)*. McCubbin et al. (1996) added items for teenagers, added a section rating valuing of the routines, and created subscales. Participants rated 32 statements describing the extent a statement about specific daily routines was true for their family (false, mostly false, mostly true, true) and how important the routine was for their family’s togetherness (not important, somewhat important, very important, not applicable). Spanish-speaking participants received a Spanish translated version we developed for this study. An expert in Spanish translated items to as closely as possible resemble the meaning rather than the English wording of each item. The authors of the FTRI report high internal reliability (32 items; α = 0.88, English version, McCubbin et al., 1996). The *FTRI*-*extent* and – valuing scores are calculated by summing item ratings. Since we wanted to compare our data to data generated by the scale’s authors we summed the same 30 items (two items were excluded in the *FTRI* authors’ normative data collection). Unfortunately, due to an error in the *FTRI* manual, we were not able to use the comparison data for the *FTRI*-*valuing* scale.

### INTERVIEWS

Participants were interviewed by a member of the research team using a series of semi-structured interview guides. Thirty-three of the interviews took place in the participants’ homes, four in a place of their choosing (their workplace or a public place), and two by phone. In total the interviews lasted 2.5–6 h, with longer interviews split into two or three sessions. An interpreter was present for interviews with participants who preferred to speak and be interviewed in Spanish. Questions from the interview guides central to this analysis are listed in **Table 1**.

**Table 1:** Questions used from interview guide.

1	What is your typical day like?
2	What did you do yesterday from the time you got up till you went to bed?
3	Do you have any daily routines that you follow or try to follow every day?
4	Are there times of day that things are likely to get stressful for you? When is that? What are you doing?
5	Are there any daily stresses which you experience?
6	What creates the most stress in your life right now?
7	When you are feeling good (not feeling good) and have a positive (negative) sense of well-being, what are you doing? Who is around?
8	Do you get any help taking care of the house or the children?
9	If you could change your day to improve your well-being what would you do?

### PROCEDURES

Caregivers were recruited from an early intervention program, clinics serving diverse children with disabilities, a project for Native American children with disabilities, and through referrals from occupational therapists. The University of Wisconsin–Madison Institutional Review Board approved the study protocol and procedures. The research team reviewed the informed consent with participants prior to obtaining their signatures. Participants were interviewed by either the principal investigator or a graduate research assistant, and were asked to complete a series of surveys that included Ryff’s *PWB* scales and *FTRI*. Interviews were audio-taped and transcribed verbatim by the research team or a professional transcriber, producing a total of 3,402 pages of data (mean transcript length: 89 single-spaced pages). Interviews with Spanish-speaking caregivers were transcribed in Spanish and English with the Spanish sections translated to English to ensure accuracy. Quantitative survey data were entered into and analyzed using IBM SPSS software (v. 20).

### MIXED-METHOD ANALYSIS

This multi-step mixed method analysis began with qualitative coding of the interview data, a descriptive analysis of quantitative data, followed by the creation of a matrix using *FTRI-extent and PWB* scores to group participants (low routine-low *PWB*, low routine-high *PWB*, high routine-low *PWB*, and high routine-high *PWB*) and finally an analysis of the qualitative differences among these groups. Given our interest in identifying ecocultural features related to well-being, we used a case-based approach to qualitative data collection and analysis (Flyvbjerg, 2013).

For the qualitative coding, first, both investigators read the entire data set to get an overview of each caregiver’s unique situation and give context to the narratives. Next, we used open coding of paper transcripts examining all data segments related to daily routines, comparing similarities and differences among cases. We specifically attended to and coded caregivers’ descriptions of features of their ecological niche. This open coding generated themes regarding caregivers’ strategies and experiences during daily routines as they negotiated them within their unique life circumstances.

To parse out differences among participants regarding well-being and routines, we subset the data creating individual vignettes, or cases, of each participant’s typical daily routine, using all relevant data segments. We organized and coded these in a series of Microsoft Excel spreadsheets that allowed us to compare participants and systematically code and refine codes using features of the Excel spreadsheet. These routine vignettes included times of day, sequences of activities, and the caregiver’s characterization of what and how things happened. Verbatim quotes that captured the caregiver’s daily strategies, emotions and difficulties were used to create the caregiver’s embodied experiences in the vignettes. The investigators recursively coded these routine vignettes separately and then met to develop consensus categories, refined from open coding, that characterized key features of routines. Specifically these categories included: the structuring of routines, degree of vigilance/intensity of daily caregiving, quality of AM/PM routines, intersections of housework and childcare, intersections of paid work and childcare, quality of dinner routines, spouse’s assistance in daily routines, children’s capacity to self-occupy, “me time,” and quality of caregiver’s sleep. We then examined variations or dimensions within the categories for each participant. To minimize bias, we completed this series of qualitative coding before calculating the caregivers’ self-ratings of their family’s adherence to routines or their level of PWB.

Next, we examined the similarity in adherence to routines between the caregiving families in this study and McCubbin et al.’s (1996) *FTRI* data. We also examined whether caregiver age, child age, family income were associated with *FTRI* or *PWB* scores*.* To determine whether caregiver well-being was related to the meaningfulness or adherence to routines, correlations between the *PWB* and *FTRI* total scores and subscales scores were computed.

**FIGURE 1:**
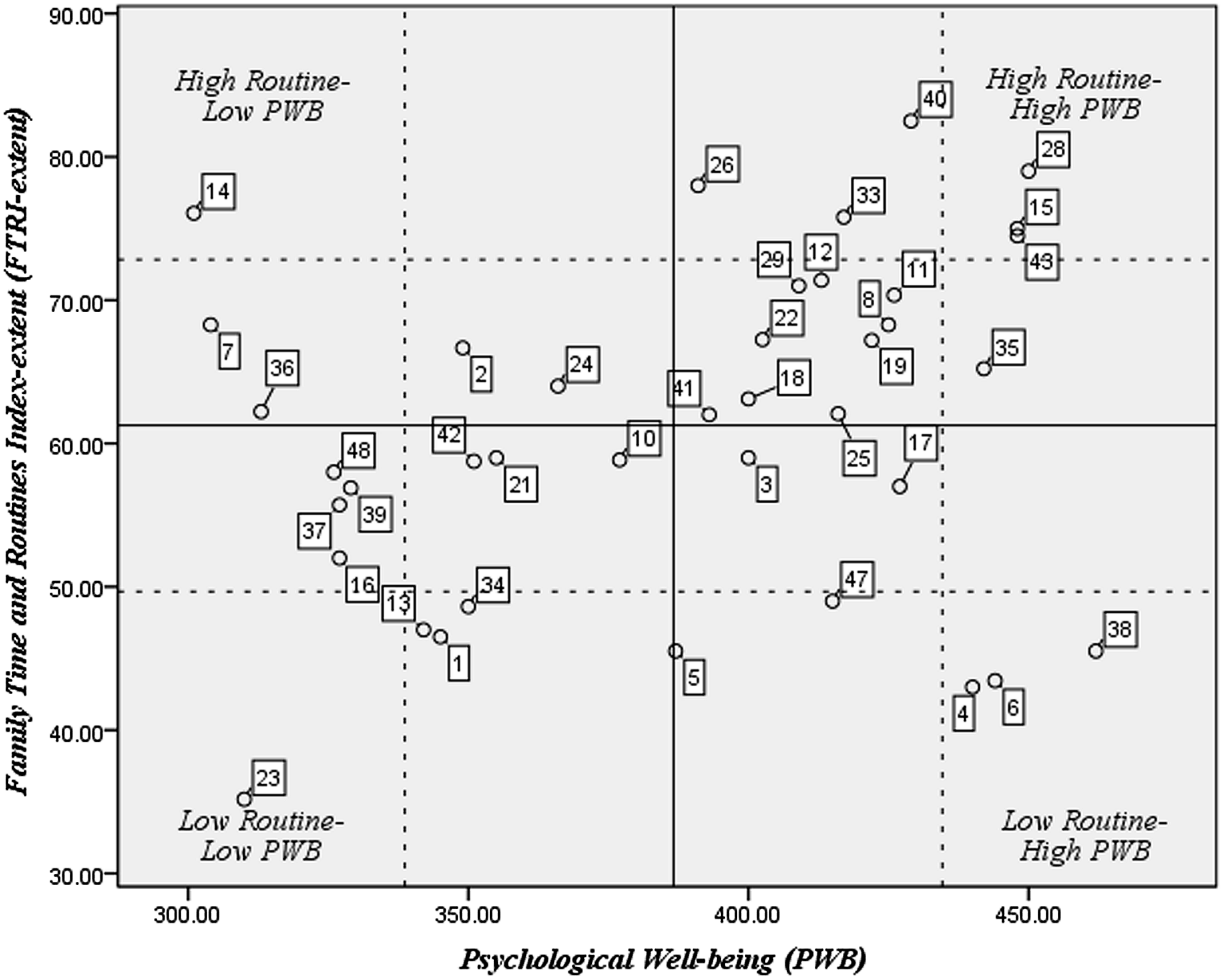
**Participants grouped by FTRI-extent and PWB scores.** Solid lines indicate means of *PWB* or *FTRI* (*FTRI*-extent* M* = 61.25; *PWB M* = 386.63). Dashed lines indicate one standard deviation above and below the means (*FTRI-extent* SD = 11.58; *PWB* SD = 48.00). Squares indicate participants’ assigned numbers.

Finally, we used the *PWB* and *FTRI-extent* scores to create a matrix for sorting participants into groups. To create the matrix, we used the *FTRI-extent* score, rather than the valuing score, because we were interested in the degree families practiced routines. Participants were sorted into groups based on their *FTRI-extent* and *PWB* scores (see **Figure 1**). The *PWB* score was plotted on the *x*-axis and *FTRI-extent* scores on the *y*-axis. We used the *FTRI-extent* and *PWB* means to delineate high and low groups. Four caregiver groups were created: (1) low routine-low *PWB* (*N* = 11), (2) low routine-high *PWB* (*N* = 7), (3) high routine-low *PWB* (*N* = 5), and (4) high routine-high *PWB* (*N* = 16; **Figure 1**). We sorted coded routine vignettes into these four groups and analyzed according to variations and crucial differences in the key features of routines previously noted. Not all of the previous categories or codes differentiated the participants in the four quadrants. For example all caregivers reported feeling tired and getting insufficient rest. Likewise, the stressfulness of morning or evening routines was dependent upon the age of the child and whether they attended school or daycare. We quantified the key features that differentiated the groups to create a table noting the percentages of participants in each group with that feature and generated a qualitative analysis comparing group differences.

Methods used to assure credibility of this analysis included team parallel and consensus coding, triangulation between data sources including comparing qualitative data and quantitative self-ratings to support findings, and the presentation of smoothed data segments to support the analysis. Participants’ identities were protected by removing family names and substituting a noun representing their family relationship in brackets. To give the reader context to the quotes, the participant’s numbers and the child’s age and disability are provided following each quote. By providing participant numbers, readers can locate each participant within the quadrants on the matrix.

## RESULTS AND DISCUSSION

We begin by presenting the quantitative findings describing the relationship of caregiver well-being to routines. Next, we have organized qualitative findings using the features of the ecocultural niche that were frequently described by caregivers: child care tasks, domestic task workload, spouse’s role in child care and household work, and family subsistence. While the eco-cultural niche model neatly separates childcare from household work and paid work, we found, as did Gallimore et al. (1989) that these features of routines were inextricably entwined and thus sometimes difficult to consider separately. Thus we provide an exemplar of an important family activity, the family dinner, which provides additional insight into the intersection of these features. We will also describe how the negotiation of these ecological niche features, as well as respite from routines, are related to caregiver well-being and routine use. Lastly, we compare the differences among the four groups on key ecocultural features.

### FAMILY TIME AND ROUTINES INDEX AND PSYCHOLOGICAL WELL-BEING

These families, as a group, endorsed nearly similar frequencies of routines as families of children without disabilities. The *FTRI-extent* group mean for our caregivers was 61.25 (SD = 11.58) compared to 63.50 (SD = 10.79) in McCubbin et al.’s (1996) sample of 304 nonclinical families. Our results showed high internal reliability similar to that reported by McCubbin et al. (1996; *FTRI-extent*, 30 items, α = 0.82; *FTRI-valuing*, 30 items, α = 0.90). The caregivers’ *PWB* group mean score was 386.63 (SD = 47.96). Neither the child’s age, parent’s age, nor family income was significantly related to the extent families practiced routines, *r*(37) = 0.11, *p* = 0.49, *r*(37) = 0.25, *p =* 0.13, *r(*37) = 1.13, *p* = 0.44, respectively, or caregivers’ well-being, *r*(37) = -0.01, *p* = 0.95; *r*(37*)* = 0.07, *p* = 0.66;* r*(37) = 0.06, *p* = 0.73, respectively.

The caregivers’ *PWB* and *FTRI*-*extent* scores were not significantly correlated, *r*(37) = 0.25, *p* = 0.13, nor were the *PWB* and *FTRI*-*valuing* scores *r*(37) = 0.04, *p* = 0.80. We also examined the relationship of the *FTRI*-*extent* score to the *FTRI*-*valuing* score and to each of the *PWB* scales. There was a strong positive relationship between the *FTRI-extent* and *FTRI-valuing* scores, *r*(37) = 0.43, *p* < 0.01. There was also a moderate positive association between the extent families engage in routines and the caregiver’s rating of *Purpose in Life, r*(37) = 0.33, *p* = 0.04. The correlation between the *FTRI-extent* and *Environmental Mastery* scale scores approached significance *r*(37) = 0.30, *p* = 0.06. None of the *PWB* scales were significantly correlated with the *FTRI-valuing* scores.

While we did not find a statistically significant relationship between caregivers’ well-being and the extent families practiced routines, there were several interesting findings. The relationship between several dimensions of the caregiver’s well-being (*Purpose in Life* and *Environmental Mastery*) and the extent routines were practiced was significant or approached significance. In the qualitative analysis, we attempt to unpack these findings as well as consider the practice of routines within the ecological niche to better understand their impact on caregivers’ well-being.

### CHILDCARE TASKS

The majority of caregivers (80%) had centripetally organized routines focused around their child with disability’s developmental needs, despite the child’s age or presence of siblings. In the case of families of children under 3 years of age, 33% of the group, this type of organization would be expected (Birenbaum, 1971; Combrinick-Graham and Higley, 1984; McCubbin et al., 1996). The caregivers used language describing this centripetal organization: “it all revolves around the baby” (Participant 10, 1-year-old child, organ transplant recipient); “Everything changes because it has to revolve around his [needs]” (Participant 40, 10-year-old child, cerebral palsy). This caregiver noted a typical reason a young child might require an intense focus: “Well, you know children think they’re the center of the universe. And it is hard not to focus on them. You cannot leave them alone for more than a minute without being amazed at what they can get in to” (Participant 39, 3-year-old, developmental delays). Specific features of their child’s disability created a greater intensity of daily demand for not just the caregivers of the preschoolers but continued for an additional 42% of families with older children who continued to utilize centripetally organized routines. The greater intensity of caring and supervision during daily routines was required for different reasons beyond preschool years often related to the child’s needs, behaviors or temperament. These included performing frequent or extraordinary feeding, medical or therapy treatments; constant oversight to foster completion of activities such as self-care or homework routines within time constraints; intense vigilance to and monitoring of the child’s behavior to prevent meltdowns by altering the routines; and meeting the child’s needs or demands for attention. Here are narrative exemplars of each of these reasons.

*Extraordinary care:* [Child] often starts retching at about 5 or... 5.35 (A.M.). So we unhook her from the feeding pump, and tell her to go and watch TV. But sometimes, what’s frustrating sometimes is there is no typical day, because it is so different from day-to-day and she’s a different child from day-to-day (Participant 23, 10-year-old, dysautonomia).

Part of it is like the therapy. [My child] has got every day a different schedule for therapy. And it is hard to find, you know, a feeding time, a lunchtime, a naptime. ‘Cause every day I have to like look at my schedule and organize what Im going to feed him when. And when I’m going out for a walk with the kids. According to that day’s schedule. So every day, I have to like rearrange. That’s kind of chaotic some times. I don’t, I would rather have something a little more regular (Participant 10, 9-month-old, cerebral palsy).

*Constant oversight:* He is supposed to eat breakfast and get his meds and get dressed before he can watch TV. It takes a lot of directing on my part to make all of that happen. And I’m trying to pull back because I find the more I direct the less he’s going to do it. I’m up there every 5 min (Participant 46, 14-year-old, autism).

*Vigilance/meltdown work:* If [child] gets up earlier, [it] really causes a problem. Because he needs the attention. Um it is fine as long as I can turn him around all morning, so that he’s not biting or hitting one of his other brothers and messing up their routine. And then uhm, so that he is not running around following me crying. You know so I end up carrying him for like an hour while you know. It’s hard putting the makeup on curling hair with one hand. But uhm. Yeah. But that’s kind of what you have to do with him. Otherwise he does, he messes up everybody else’s routine (Participant 34, 3-year-old, autism).

*Demands for attention:* If he needs something, you have to literally stop what you’re doing and look at him and try to figure out what he needs. He knows a few signs. We’ve learned just a few signs. That’s what we’re working on. It’s the only way he has to communicate (Participant 4, 2-year-old, dyspraxia).

She wants all of my attention, all of my heart. She wants everything and she will maneuver things to try to make that happen which disrupts everything in the household (Participant 5, 2-year-old, reactive attachment disorder).

Caregivers described how this concentrated focus on the child and the necessity of being immediately responsive curtailed their control of the daily schedule and activities. Not being able to plan when activities would occur or to complete activities they’d started could be distressing and impeded the performance of essential household tasks. The perception of control, as measured by participants *Environmental Mastery* scores, differentiated our groups: 12 caregivers in the high routine-high *PWB* group had *Environmental Mastery* scores above the mean, one in the high routine-low *PWB* group, four in the low routine-high *PWB* and two in the low routine-low *PWB* group. The sense of control over one’s daily life was an important factor in caregivers’ well-being.

Of the 20% of caregivers whose narratives suggested that their children could participate in regular routines, one had a toddler described by his mother as “flexible” in regards to routines, and the other seven had children over 10 years of age, most with physical disabilities. These parents did not describe their children as demanding nor as having behaviors that needed more intense management.

For most of these families it was not only the child’s age but also specific features of the child’s disability that required the continuing centripetal organization of the family’s schedule. The constancy of attention and caring increased the demands on the caregiver, for many taxing their capacity to be responsive in relationships. This intensive focus on the child’s needs had a ripple effect on the caregiver’s domestic workload that could be blunted by support from the spouse or other family members.

### DOMESTIC TASK WORKLOAD

With the centripetal organization of routines, caregivers often noted the tensions between caring for the child in a way they valued and completing the housework they deemed critical: meals, dishes, laundry and some level of household cleanliness. They employed strategies including lowering their standards for the quality of household management, using fast foods or quickly prepared meals, multitasking by weaving housework and childcare, alternatively “carving out time” when their children were occupied or sleeping to do housework or relying on support from their spouse or another family member. This first mother found time after the last of her three children was asleep; the second mother also carved into her sleep.

Although, I think an almost 5-year-old [child] should be going to bed a little bit earlier than 9:30 especially when Mommy would like to go to bed earlier than 9:30... And then I did whatever I needed to do and went to bed which may have been finishing cleaning the kitchen. I mean at the point I’m not going to fold any more laundry... you know those are the two big – laundry and dishes. Those are the two big things that... And clean the toilets. You know and pretty much everything else can wait. But those three things have to be done... and fix food. But as far as cleaning I consider kitchen, laundry, and toilets to be the items that can’t wait (Participant 13, 21/2-year-old, speech delay).

So I found, during the day, the housework cannot get done very easily. I mean there are a few things that I can do with him around but a lot of it has to wait until he is asleep. So if I do that and then if I have anything that I want or need to do then I stay up late to do that. So it is not unusual for me to go to bed at midnight or later (Participant 3, 1-year-old, conditions associated with prematurity).

This mother described the complex mental calculations she considers in balancing out her daily demands and life roles to weigh toward providing quality time with her children:

I’m defeated by myself cause... I’ll just say I cannot…first of all they are going to hang on to my ankles while I try to cook, then they’re not going to eat it, so I’ll end up saying “Oh screw it we are going to Carl’s Jr.,” because then I won’t have dirty dishes and they’ll eat it and we’ll be done. So I constantly feel defeated in the house as far as cleanliness and I constantly feel defeated about the nutrition and food stuff. ‘Cause I just can’t. I’m trying to be a PhD, and I’m trying to be a therapist, and I’m trying to be Susie Q homemaker, and I’m trying to be totally quality time with the kids and quantity time. I mean major time and, the relationship. And it’s just impossible is what it boils down to. So I sacrifice the mess and the food and all that to be with the kids and try to keep my own thing going over here (Participant 18, 5-year-old, autism).

In making these choices, the caregiver often considered the children’s needs first, then their partner’s desires (if they had a partner) for desired level of cleanliness or meal preferences. While cognizant of these desires, the mothers, and one caregiving father, were often not able to be responsive to these desires. The caregiver’s decision making around these issues was often influenced by the energy the caregiver had at that time of day and the emotional work required to foster peace and harmony in the home. This mother responded to a query about when her day was likely to get stressful:

The afternoons, when it’s towards the end of the day and I’m tired and the kids are making a lot of demands and I feel like I have n’t necessarily accomplished as much as I want to that day. And it’s starting to get really hot, we do n’t have air conditioning and it’s stuffy in here. And I have tons of things to do and they’re making demands and they’re not listening to me. And I feel like I’m doing everything for everyone else and they’re not behaving and that’s not fair... then I feel bad about it, cause I don’t want to say it to them, cause I do n’t want them to feel guilty. This is my choice to do those things. Then [my husband] comes home and the first thing he does when he walks in the door is I let him know how badly behaved they are and so he’s like “Oh God, it’s good to be home.” (Participant 22, 6-year-old, autism).

This mother described an emotional overload – doing for everyone else while still not feeling effective in parenting with sensitivity, and failing to create a “haven” for her husband to come home to. Her fatigue at this time of day, and competing care and household demands, impaired her from being as responsive as she desired to her children and husband. She was not alone in falling into an afternoon doldrums when all of the family came together at the end of the day. This was very common among many of the families, where dinnertime was challenging. This is similar to the 6 o’clock crash noted by researchers in families of adolescents where emotions could go astray as families arrived home and competing family needs mounted (Larson and Richards, 1994).

In addition, this frequent fatigue at the end of the day disrupted couples’ intimate relationships. All of the caregivers who mentioned this issue noted they were simply too tired. Married Mexican American mothers reported that their husbands often told them they were neglecting their wifely duties. These mothers noted that their children’s needs came first, leaving the mothers feeling guilty about this perceived failing.

### SPOUSE’S ROLE IN CHILDCARE AND HOUSEWORK

Given the intensity of caregiving demands, and competing demands between care and housework, the spouse’s support was often critical within daily routines. Although 87% of our caregivers were married, there was wide variation in the support available to the caregiving parents in this study. Here are two narratives describing the range of support spouses offered.

With the house he helps cook meals. I would say we’re 50/50 on cooking meals, um he probably does 90% of the laundry or say 75%. We both hate dusting (laughter). If we could hire somebody to dust, life would be better. Um as far as like meals and cleaning up, you know doing dishes and stuff that’s probably 50/50. Um we probably do 50/50 on that. Like I said with the laundry, he takes care of most of that. Um picking up the house probably is more me than him just because of the times. Although at night he generally tries to pick up but sometimes I say “Do n’t worry about it, I’ll do it in the morning,” because you’ve got to recognize he needs to have some time for himself too... And he takes care of [child] predominantly in the evening [while I work] (Participant 8, 21-month-old, oculomotor dyspraxia).

He got mad because he did n’t want to take off work to watch [child] while I was taking her to treatment but he did n’t want my mom to be here. He wanted me to schedule things like when [child] was at pre-school. Well, she naps in the afternoon so it’s very difficult to take her to like speech therapy where she will just fall apart. Um so dealing with him on any scheduling issue and particularly regarding her with the therapies and...You know what’s good for me, what’s good for her does n’t match [his schedule] (Participant 5, 21-month-old, reactive attachment disorder and medical condition).

There is a clear difference in the support each of these caregivers experienced and in turn how this influenced the quality of their marital relationships. Being in it together or working as a “tag team” was described by several families (Maul and Singer, 2009), especially for several who had a child with special needs as well as typical children, or who had two children with disabilities who both demanded one-to-one attention. Around 28% of spouses helped with childcare when they arrived home from work or on weekends, and 10% helped with cooking dinner on weekdays or weekends.

### INTERSECTIONS OF CHILDCARE, DOMESTIC WORK AND SPOUSAL SUPPORT: THE FAMILY DINNER

The family dinner is a time when the childcare, the domestic workload and spousal support come together in an essential daily task. Family dinner has been viewed in U.S. culture as an indicator of the stability and health of the family, and a means to support children’s emotional development and wellness (Gibbs, 2006; Fruh et al., 2011). For our participants, this one item, the “family eats at least one meal together daily,” served as a key indicator for the extent to which families participated in routines overall. None of the participants falling one or more standard deviations below the group mean *FTRI-extent* score consistently ate dinner together as a family, whereas all but one of the participants one or more standard deviations above the group mean *FTRI* score consistently ate dinner together as a family. Those within one standard deviation of the mean mostly ate dinner together as a family, with four exceptions: two whose spouses worked shift work, one whose daughter was tube fed on a strict schedule, and one whose children’s high demand for vigilance significantly altered the evening routines.

For Mexican-American families in our study, the form of family dinner differed. Several Mexican American mothers described cooking dinner for their children in the early afternoon and having another meal for the adults in the later evening. Though the family was not necessarily eating together, they were still creating purposeful family time.

In fact, I only keep them company. I eat something light because I have already eaten because for me 6:00 pm is very late. My daughter and I, we eat together. But always, since it’s a tradition in our family, we all sit together at the table. So we keep them company by eating cereal or fruit, but we do sit with him (Participant 40, 10-year-old, cerebral palsy).

The togetherness of family dinners, when all family members have a need to be met, is more difficult if centripetally organized around the child with a disability. The need for and availability of vigilance appeared to be instrumental in the quality of dinner routines. Caregivers who were able to be less vigilant about childcare during dinner and dinner preparation were more likely to consistently and effectively orchestrate family meal times. In families in which the children could not be left alone, the spouse needed to play an active role in dinner preparation either cooking or watching the children.

Dinnertime was more likely to be stressful and less likely to be a time the whole family was together when the child could not be left alone and the spouse did not help. Caregivers in this situation, who desired consistent family dinner routines, were highly vigilant and multitasked to accommodate the multiple needs of their family. For example, this caregiver describes juggling childcare, cleaning and providing dinner for her husband.

I’m often feeding them while I’m fixing something for my husband and I. We have a counter in the kitchen that they can sit at and I can – they’re on the other side and I can be on the kitchen side and so that sort of facilitates feeding them and working in the kitchen at the same time. So as a result – I mean at this point we do n’t really sit down as a family. They’re kind of done and then my husband eats and he’d just rather read the newspaper so I rare – I sit down for a little while sometimes but mostly the kids are done and so, you know, I kind of eat and clean up and manage them (Participant 13, 2-year-old, speech delay).

The tradition of the family dinner was valued by these families, however, not all of them were able to orchestrate a meal that included everyone or met their standards. This was sometimes due to the child’s needs but just as often due to the father’s shift work schedule. In some cases where it was too difficult to orchestrate, caregivers reverted to a functional approach, making sure everyone was fed, even if the quality of food was not up to their standards. Still the ideal of a family meal was associated with higher caregiver well-being. Dinnertime was most likely to be positively related to caregiver’s well-being when (1) childcare and meal preparation were segregated, (2) the spouse helped with one or the other, and (3) everyone was present for the meal.

### FULL-TIME AND PART-TIME WORK OUTSIDE THE HOME

In addition to caregiving, some mothers worked for pay part-time (23%) and some were employed full-time (23%). Choices to engage in paid worked were based on parenting values, the quality of services available for the child during the day, the flexibility of work hours, and the caregiver’s desires for work. Some parents described their choice to have children and their desire to raise them, especially those under five years of age. In some cases caregiving parents did not feel they had a choice, since the kind of care their child needed was not available in daycare, and so one parent stayed home. This was especially the case for medically fragile children or those with complex needs. If parents could find childcare, it had to be of a sufficient quality that eased their worries, and their work hours had to be flexible enough to allow them to leave at a moment’s notice. Specific features of their child’s needs, such as frequent illness related to their chronic condition or severe behaviors that required the parent to pick the child up from childcare or school, or intensive case management demands to support the child’s continued service provision could curtail the caregiver’s opportunities for employment. Alternatively, some caregivers worked part-time to supplement the family income and relied on childcare or family members to provide care. Some caregivers’ preference was working full-time. Others desired to return to work, but were not able to because of the frequency or nature of the demands of their child’s care, even when the child was enrolled in school. The tensions between sufficiency of care, sufficiency of family income, and desires to work were complex and managed differently by caregivers. These narratives illustrate the tensions and variations among caregivers:

I think if I had to work as well that would be very stressful I would not be happy. I would n’t be able to you know give the care to my children that I want to. So you know being able to... have this time with them to care for them that contributes to my well-being (Participant 13, 2-year-old, speech delay).

I have to go back to 40 h a week from a monetary standpoint. I started out at thirty-hour week when I started prepping for the IEP [Individual Education Plan]. And I knew the services were going to change... And then they did n’t offer a lot of therapies in the evening any more. So if my son was going to be attending therapies, my husband and I were going to have to take work off to take him (Participant 34, 3-year-old, autism).

If I had more [nursing] time... I would like to be able to find a job that professionally interested me, was flexible enough, obviously, to be able to care for [child] but yet where I was able to meet people. I think one of the things that was the saving grace when we were in Indiana and I was working full time, and we had more nursing, was when I got to work, well, I always thought about my kids and my husband, it was never out of my mind completely, but I was able to really able to focus on the task at hand. And, um, I think that was really freeing (Participant 11, 1-year-old, medically fragile).

Sometimes I figure I’ll [work] at night but by the time we get them to bed, or if we get them to bed, when I leave a little early it stresses [child] out and it stresses her out and [my husband] gets dumped with them stressing out. It’s just... I do n’t have any time to myself. I think I realize now I think that’s why I’m enjoying working because even though it’s, I’m accountable to someone else, it’s really time for me. It’s like a haven (Participant 22, 6-year-old, autism).

Occasionally I swing over to... I just have to be a stay at home mom, you know, and just do a good job with this. But if I, when I’m in that state where I have nothing else going on I very quickly just get really depressed. Because then I do n’t even get out at all. And... I do n’t have that sense of being connected to these larger things... I do have a good mind and I need the stimulation. I mean frankly I just need something (Participant 18, 5-year-old, autism).

I would not be a good mother if I was home with him 24 h a day. I just can’t do it. I need to get out and be with adults and do something that is more rewarding for me than changing diapers. So I still have that. I’m not working quite full time. And I’m not taking on a lot of the projects that I used to... But at least I still have that other dimension to my life. I think it’s more that he’s never far from my thoughts. And you know the first thing I do when I leave the school is turn on the cell phone in case the school has to call me between the time I reach school and the time I get to the office. I look at things differently because there’s a child in my life (Participant 39, 3-year-old, developmental delay).

The child’s needs, as a continuing centripetal force, still drove the caregiver’s choices of jobs based on the number and timing of work hours. It was also evident that engaging in work provided caregivers with a place to have greater control, measurable successes, adult company, and opportunities to pursue their own interests. What was surprising in this data was the sense of paid work as a haven or place to feel freedom from caregiving worries.

While some research has demonstrated that more hours of maternal employment may diminish frequency of family routines in typical families of preschoolers (Anderson, 2012), for these caregiving families of children with disabilities, maternal paid work provided respite from demands, social contact with adults, and a place to demonstrate competence. Employment for these mothers counter-balanced the challenges of caregiving, which could be repetitive and less rewarding, and gave them an opportunity to feel effective and productive. Hyde et al. (2004) noted the powerful role the child’s characteristics play in the experiences of work and home life for a mother. They found that a typical child’s difficult temperament predicted mother’s work outcomes and that this was mediated by perceptions of parental competence (2004). Warfield’s study of caregivers of young children with disabilities’ work interest, family support and parenting stress noted the complex relationship between these variables. She found that parent demands and family support were good predictors of parent stress, but that once these variables were controlled for, being interested in one’s work was associated with lower perceived parenting stress (Warfield, 2001). While interesting work served as an additional stress “buffer,” at the highest level of parenting demands this was no longer true. Olsson and Hwang’s research likewise noted the positive relationship between employment and well-being for caregivers of children with intellectual disabilities. It could be that as Sheldon and Niemiec (2006) suggest, the happiest people have a balance between autonomy, competence and relatedness in their lives (Ryan and Deci, 2001). It seems that in this study, greater balance among these features, countering the strains of caregiving, could be achieved via paid work.

Still not all mothers believed they could be employed even if they desired it. For example, parents of medically fragile children had no alternatives for the intense medical care they provided in-home day and night. While some families had daily nursing care, it was often insufficient and what little respite services were provided were used to manage household tasks or do errands. Similarly families of children with autism or cerebral palsy could often not find competent caregivers to babysit the children. Thus the children’s needs for care of a sufficient quality drove the choices that surrounded family subsistence and opportunities for leisure.

### RESPITES FROM THE ROUTINE: “ME-TIME”

Being well rested was essential for the caregiver to be responsive to children and manage the daily demands. While the morning could be stressful due to the time pressure and constant supervision to get children through morning routines in time for school, the afternoons were most often described as a difficult period in the routine. For example, “In the evening, I’m tired and I have less patience. And as the evening wears on and [my child] is full of energy. I have declining energy and I get really irritable in the evening” (Participant 38, 3-year-old, developmental delay). Both dinner and bedtime could be difficult when caregivers were tired. “Meal times are bad and evenings are bad. It’s just normal. Evenings... when you’re... Suppertime, they’re getting tired, they’re hungry. And then you got to work on bedtime. And being alone most of the time because [my husband is] in bed sleeping, or else already gone to work” (Participant 4, 2-year-old, dyspraxia). Caregivers valued time *away* from caregiving and family demands; this occurred as small breaks during the day.

Why I stopped working, was to make sure he gets as much help as possible, so…when you’re *concentrating* on that, what I had to learn to do was try and find some little pockets of time to just... relax and have time for myself. I went to therapy for a little while “cause I was so stressed... that’s what the therapist was *saying*.” You need to have some time (Participant 1, 10-year-old, autism).

That’s the main thing I feel I need right now. To become more balanced... breaks from the kids are definitely necessary. Sometimes I get enough, and sometimes I don’t... the past week I don’t think I’ve had enough breaks. Last night I was just thinking, oh, I really need a break. I had a headache and it’s really hard when you have a health problem because you can’t really just lay down and rest... I did lay down for a while, but then [child] woke up. I had to feed him and stuff. So you have to kind of go on anyway (Participant 6, 2-year-old, cerebral palsy).

Caregivers used phrases like “me time” and “my time” to describe integral parts of their days. Me time was restorative, autonomous time spent away from the responsibilities of caregiving when their children were safely and sufficiently occupied. Me time happened either when children were asleep (before waking, during naptime, after bedtime), following a shift of childcare duties to a willing and able family member or professional, during school hours, or when daycare was available at the gym.

When the caregivers had opportunities to leave the home, they went to social engagements with other adults to play cards, meet friends for coffee, sing in the church choir, exercise or dance at a Powwow. Some caregivers took walks or bike rides. One mother took walks to “mentally try to clear [her] mind” (Participant 13, 2-year-old, speech delay). This mother with a high level of well-being used regular exercise:

I exercise more than I used to because it’s a good way for me to get away and have little time to myself. You know, before I had plenty of time to myself and at least now I’ve got guaranteed 3 or 4 h a week where I can exercise and have the kids taken care of by somebody else (Participant 12, 2-year-old, speech delay).

Me time within the home typically consisted of solitary restful activities and diversions such as reading, listening to music, watching television, sewing or crafting. While some caregivers engaged in me time while someone else was caring for the child, this was less common than using the time when the child was asleep. Parents of young children in particular capitalized on using naptime for a break.

But usually she’s down to one nap a day now. And, ah, that gives me a couple of hours to myself. Um, you know, where I can do laundry or do a few house cleaning. But I also always take time to, you know, maybe read the paper and just sit down for 5 min and try to catch my breath (Participant 10, one-year-old, medically fragile).

Some participants used the interval between the child’s bedtime and their own bedtime - or in one case, the time before the child woke up - for me time. Caregivers often traded sleep for time to themselves. After her daughter went to bed, this mother stayed up to read: “That’s my relaxation hour... I get to bed anywhere between 11:00 and, if it’s a really good book 1:00” (Participant 15, 11-year-old, cerebral palsy). Given high demands for care and low levels of support some participants could not find me time. In these cases, caregivers found pockets of time while continuing to be responsible for caregiving. One mother of three young children capitalized on times her children were engaged in play to take brief time to herself: “When they’re busy, I’m watching TV. That’s my only break. I get breaks to watch TV and have my cigarette” (Participant 14, 9-month-old, cerebral palsy). She also noted the diminished quality of these breaks: “I don’t know how to relax anymore ‘cause I’m always worried about what the kids are doing. Even when they’re sleeping. I’m always worried.” This diminished me time was characteristic of many of the participants with low *PWB* scores.

Caregivers desired more time for themselves. When they were able to find time, they often used it in passive leisure, most often watching TV or reading. Few participants exercised. Regular exercise was difficult to orchestrate in the schedule unless someone provided a break from childcare. In contrast, passive leisure occurred most often when the children were occupied or asleep and still in their care. In addition for most caregivers, with the exception of the exercisers, me time was infrequent, occurring weekly or monthly. TV was the only “me time” that happened with more frequency during the week.

When available, leisure me time was most often solitary, was passive, and occurred in the home since caregivers often could not leave the home. The opportunities for me time were usually determined by the available spousal support and child characteristics.

### DIFFERENCES AMONG GROUPS

The orchestration of daily routines, while managed mainly by one family member, is in fact the result of a family group interaction. Between these routine-*PWB* groups there were differences in the capacities of the child, support of the spouse, and energies and efforts of the caregiver that contributed to the sustainability and quality of the family’s daily routine (**Table 2**). In both of the high routines groups, at least one-third of the children were able to follow a routine, so caregiver’s efforts in most of the families still required concentrated and intense efforts to sustain daily routines. Few children in the low routines groups followed regular routines easily. This was one essential difference between the high routine-high *PWB* and low routine-high *PWB* groups: children of the low routine-high *PWB* had great difficulty or needed constant oversight to follow and participate in daily routines.

**Table 2:** Comparison of number and percentages of caregivers with key ecological features by group.

Key features	High routine-high* PWB*, *N* = 16	High routine-low *PWB*, *N* = 5	Low routine-high *PWB*, *N* = 7	Low routine-low *PWB*, *N* = 11
Child self-occupied in an acceptable way for a short time	11 (69%)	3 (60%)	2 (28%)	6 (54%)
Child followed daily schedule without intense oversight	5 (31%)	1 (20%)	0 (0%)	1 (9%)
Above mean score on *Environmental Mastery Subscale*	12 (75%)	1 (20%)	4 (57%)	2 (18%)
Spouse/others helped regularly with childcare or household tasks	10 (62%)	1 (20%)	3 (43%)	5 (45%)
Family ate at least one meal together daily	13 (81%)	5 (100%)	2 (28%)	7 (63%)
Spouse/others helped during dinnertime	4 (31%)	1 (20%)	2 (28%)	3 (27%)
Worked full or part-time work outside home	12 (75%)	3 (60%)	2 (28%)	2 (18%)
Engaged in active “me time” activities	5 (31%)	2 (40%)	1 (14 %)	3 (27%)
Engaged in passive “me time” activities	5 (31%)	2 (40%)	6 (86%)	6 (54%)
Engaged in no “me time”	6 (38%)	1 (20%)	0 (0%)	2 (18%)

Spouses in the high routine-high *PWB* group were more likely to provide assistance in childcare and household tasks. The least support was provided to caregivers in the high routine-low *PWB* group. In this group caregivers expressed dissatisfaction with the help the husband provided or could not count on help due to irregular hours or shift work. In the low routine-low *PWB* group, there was a variety of different family situations: several caregivers did not have spouses, some families followed very traditional roles in which mothers were responsible for the majority of household management, and several families had multiple children with special needs requiring a team effort by both parents. The low routine-high *PWB* group, though small, was particularly interesting. The lowest raters of the routines in this group, those more than one standard deviation below the mean, had to work around unsupportive husbands’ routines or a shift work schedule while managing family life. For example, in one participant’s family (5) this ranged from disruption (“he disrupts any type of routine we have... he has no concept of what I’m doing with them.”) to not coming to dinner when it was ready, to not helping with difficult bedtime routines for his two children, and putting his sports schedule first. These family units’ schedules were fractured, in that the husband’s preferences and plans for leisure or school schedule came first, or that the shift work disrupted family life. The two other families in this group, also one standard deviation below the *FTRI-extent* mean, were atypical as well. In one the caregiving mother was also the major wage earner and had great difficulty organizing reasonable family bedtimes. Her husband kept his own schedule with late bedtimes and risings. In the other, the parents were managing two challenging boys with autism that were very capricious day to day.

Another difference among the groups was the number of families able to dine together. In some ways this was symbolic of their ability to come together as a family, a barometer of the family’s sustainability of daily routines. The high routine-high *PWB* group had the most families who had meals together regularly. The other high routine group also rated it mostly true or true that they had one meal together daily, but from their narrative descriptions it was clear that often the husband was absent from meals. Interestingly again, the low routine-high *PWB* group had the fewest families sharing a meal. Reasons for this again reflect the spouse’s choice to not be available after work or the influence of a shift work schedule where the mother was most often on her own.

In regards to the primary caregiver, having paid work, if they desired it, was associated with higher levels of well-being. In contrast to other findings that work could spillover in a negative way or sap energy, the caregivers experienced a positive spillover from work (Bronfenbrenner, 1986). Work seemed to blunt the social isolation and give caregivers a sense of control and ability to use their talents. Despite increasing their workload by adding an additional set of demands, work, either full- or part-time was for some a “haven” from caregiving demands. Thus the group with the most working caregivers had higher well-being. It may also be that the additional income reduced the family’s financial insecurity, and thereby bolstered the caregiver’s well-being. In addition, caregivers in the high well-being groups were more likely to have a sense of control over their life circumstances indicated by their higher scores on the *PWB Environmental Mastery* scale. This greater sense of mastery may be linked to their ability to find and keep paid work that was flexible enough to work with their caregiving.

Lastly, there were trends in well-being related to the ethnicity of our caregivers. Interestingly, all of the Native American mothers and all of the Hispanic married caregivers were in the high routine-high *PWB* group. These mothers’ ethnic practices and beliefs appeared to confer some advantage in their view of their lives and daily routines. Hispanic caregivers who were single, or who described a significant marital challenge, fell into or were immediately adjacent to the low routine-low well-being group. For these mothers, the lack of support or marital discord, and for those with a low income or without a husband, the financial challenges made daily life much more difficult.

What allowed caregivers to create sustainable routines while also maintaining their well-being? Caregivers who worked full- or part-time; had a supportive spouse who provided instrumental or emotional support; had children more capable of participating in daily routines; had a better sense of control of their life circumstances; were able to orchestrate regular routines, as indicated by the family meal; and who were able to find breaks from care either in work or leisure had the highest levels of well-being. Alternatively, stay-at-home caregivers who had less available spousal support or no husband, had children who had more difficult or capricious behavior day-to-day, had little perceived control of their life circumstances, had difficulty sustaining regular satisfying routines, and had limited breaks from caregiving had lower well-being.

## IMPLICATIONS FOR PRACTICE AND LIMITATIONS

Health professionals and educators are increasingly interested in inserting developmental and health-promoting interventions for children with disabilities into the family’s daily routines (Dunst et al., 2000; Fiese et al., 2013). The impetus for this effort is evidence that the family’s everyday practices impact health and developmental outcomes of its members (Bronfenbrenner, 1986; Christensen, 2004; Crespo et al., 2013). For example, early intervention has been found to be more effective when embedded within existing routines rather than created as standalone activities (Cheslock and Kahn, 2001; Lucyshyn et al., 2007). However, altering personal behaviors is proposed to be the “single greatest opportunity to improve health” (Schroeder, 2007, p. 1222). In many cases for both individuals and families, the adoption of evidence-based interventions may not occur as desired if these interventions do not fit within customary daily routines. For this reason targeted resources are being put forth to increase the adoption of health-promoting lifestyle and behavioral changes (National Institutes of Health, Office of Behavioral and Social Science Research [NIH], 2013).

Situating interventions within a family’s daily routines can only happen if professionals move beyond a simplistic understanding of daily routines as mundane and ordinary sequences of events that may easily be altered, to a more sophisticated comprehension of the multi-layered intricately constructed web of chosen activities and practices that must be sustained with daily efforts. Weisner et al. (2005) argue that sustainability of routines should be considered a core outcome for families of children with disabilities. It is this sustainability, this regularity that is believed to provide recurring opportunities for health- and development-promoting activities.

Designing tailored family-based routine-focused interventions should ideally address the specific issues impeding the positive flow of family life, and reduce the intensity of caring demands. Interventions aimed at fostering the child’s, spouse’s, or caregiver’s skills to participate in certain daily life activities provide avenues, when understood within the larger context of family life, to improve the caregiver’s ability to sustain functioning daily routines. Targeted interventions to improve the child’s capacities may not only benefit the child but also the family. Intervention that fostering the children’s ability to participate in routines by developing specific self-care or leisure skills could create “pockets of time” during difficult periods in the daily routine and thus ease the intensity of demands placed on the caregivers. Eisenhower and Blacher (2006) found that caregivers whose children had more skills had better well-being. Likewise, timely assistance from spouses or other family members at critical junctures can be highly valued (Larson, 1996). For many of these caregivers, me time in both leisure and work was essential to counterbalance to the daily stresses. Caregivers could benefit from lifestyle coaching to identify instances where me time may be inserted and sustained within their daily schedule to bolster wellness. A nuanced understanding of family routines can be leveraged by practitioners to situate interventions within critical time periods, to better align them with family life, and to promote wellness rather than increase caregivers’ demands and burden.

Though tacit in the narratives, ecological features of the macrosystem (laws. economics, culture) and exosystem (parents’ work, schools, neighborhoods, extended family) clearly impinged on the family’s capacity to orchestrate daily routines within the home and in turn on caregiver’s well-being. For example, the U.S. early intervention service delivery system is mandated by law to follow family-centered principles in service delivery. Yet in this study, caregivers found these in-home interventions intrusive at times and found the irregularity of service provision disruptive to daily life. Service agency policies could address these issues by simply routinizing the child’s therapy schedule. Similarly, second or third shift work or extended work schedules increased the burden on stay-at-home caregivers to simultaneously manage childcare and pressing household tasks (such as dinner) without spousal support, and reduced the likelihood that all family members could gather all together at least once per day. Asynchrony in the schedule due to shift work or the misalignment of family member’s activities impacted family routines and diminished opportunities to spend time together as a family unit. In addition, caregivers who wished to work or participate in health promoting leisure were limited by the childcare available in their community. There was a desperate need for reasonably priced specialized childcare that could care for children with complex medical and developmental needs and fluctuating health. Expanding the options and supports available for caregivers whose children’s needs appear to exceed the capacity of typical childcare and school settings and thus require at-home-care is warranted

There are limitations to our study. While the sample size was sufficient for our mixed methods purpose, with a larger sample size we may have demonstrated more robust relationships between ratings of caregivers’ well-being and the extent they practiced routines. In addition, we chose to study the primary caregiver and thus these findings largely reflect the mothering perspective. Additional insight into the negotiation and management of domestic tasks and childcare workloads could be gained by a didactic approach, interviewing both parents together. Future research could consider this idea of balance of the caregiver’s autonomy, relatedness, and competence. For example, this could include examining the balance between levels of environmental mastery, positive relations with others and perceived competence in managing family and work life that are related to well-being and health. In addition, the ethnic differences noted in our study suggest that some cultural beliefs may also confer advantages to caregivers; these should be examined in future work. Given the complexity of the multiple factors that contribute to caregivers’ well-being, a more simple metric that identifies areas of strength and need could be used by health care providers to design and target intervention that promote well-being for home-based caregivers with the greatest needs.

## CONCLUSION

Using an ecocultural lens allowed us to consider how cultural and institutional features of the families’ unique niches intersect to impact the caregiver’s capacity to sustain daily routines when parenting children with disabilities. Gallimore et al. (1989) noted that an ecocultural niche approach veers from an emphasis on pathologies of caregivers, focused on depression and stress, to descriptions of successful accommodations that bolster positive family functioning. While caregivers in our study described challenging daily life circumstances, many were able to construct daily routines aligned with their parenting values, albeit heavily weighted to be responsive to their child’s needs, that were tailored to their available personal and economic resources and that were associated with positive well-being. Their narratives provide clear and compelling data on how social and cultural forces play out in family life. Our approach and analysis led to some expected findings, as we have described, as well as some surprising ones. Previous research had not elucidated the multiple functions of paid work for caregivers, serving to provide meaningful respite as well as enhancing self-perceived competence. In addition, while many studies suggest ethnic minority status and low incomes diminish parenting sensitivity and family functioning, using an ecological approach and a focus on positive functioning, we found ethnic minority status to be associated with higher levels of well-being. Thus a mixed method approach, framed in ecological theory, allowed us to plumb the complexity of family routines as related to caregiver well-being, and to generate new insights and potential avenues for intervention.

## Conflict of Interest Statement

The authors declare that the research was conducted in the absence of any commercial or financial relationships that could be construed as a potential conflict of interest.
